# Medico-legal aspects of congenital heart diseases in buying and selling of pets

**DOI:** 10.14202/vetworld.2017.130-135

**Published:** 2017-01-29

**Authors:** Annamaria Passantino, Michela Pugliese, Valeria Quartarone, Natalia Russo, Roberto Bussadori, Bartolomeo Guercio

**Affiliations:** 1 Department of Veterinary Sciences, University of Messina, Polo Universitario dell’Annunziata, 98168, Messina, Italy; 2 Veterinary Practioner, Catania, Italy; 3 Veterinary Clinic Gran Sasso, Milano, Italy

**Keywords:** companion animals, congenital defects, heart diseases, purchase, redhibitory defect

## Abstract

**Aim:**

The veterinarian should be able to assess congenital and inherited malformations such as heart defects because they may be object of legal disputes. In this study, the authors report some cases of congenital heart defects in pets (dogs and cats) to clarify whether or not they may be considered a redhibitory defect.

**Materials and Methods:**

A total of 28 medical records of pets referred with suspected congenital heart disease were examined. All patients aged between 3 and 24 months underwent clinical examination, chest X-ray examination, electrocardiogram, and echocardiography and angiocardiography when necessary.

**Results:**

Congenital heart diseases or associated cardiac malformations were confirmed. Considering the above congenital diseases as redhibitory defect and the rights of the owners from a strictly legal viewpoint, 9 owners demanded an estimatory action and 11 a redhibitory action; 1 owner decided to demand the reimbursement of veterinary expenses because the animal died; 7 owners took no legal action but requested surgical intervention.

**Conclusions:**

Until more appropriate and detailed legislation on the buying and selling of pet animals is put in place; the authors propose to include in the contract a temporal extension of the guarantee relating to congenital heart disease, which can often become evident later.

## Introduction

In pets (dogs and cats), there are several heart diseases of proven or suspected hereditary origin, both congenital and non-congenital, which may constitute a vice in buying and selling. Early recognition is of great importance not only to apply appropriate medical or surgical management and provide an accurate prognosis [1] but also to avoid litigation.

In fact, cardiovascular defects may lead to civil lawsuits if the lesions are congenital, but not easily recognizable, or when the lesions are hereditary, but tend to became manifest only after some time (more than 12 months after the date of purchase, i.e., after the vice-free-guarantee period has expired) [2].

Moreover, diagnosis is frequently not made because of the owner’s delay in consulting a veterinarian or because a general practitioner veterinarian does not appreciate the significance of subtle clinical findings.

In continuation of a previous study on this issue [2], this paper was designed to focus on the medico-legal aspects concerning the buying and selling of dogs and cats affected with congenital heart diseases that may constitute vice in these animals. For this purpose, the authors report some cases of congenital heart diseases in dogs and cats to clarify whether or not they may be considered as redhibitory defect.

### Legal framework

In the last decade, the status of pets (cats, dogs, rabbits, etc.) has changed; they are becoming more important as family members, almost like children. Despite this status, they are categorized as mere goods [2] or commodities during the sale process (purchase). According to the law, animals are considered a form of personal property [3]; they are goods to be bought and sold, acquired, and maintained [2].

The term “goods” is meant to afford buyers and sellers certain rights and responsibilities in the transaction.

In Italy, the civil code (article 812) considers animals as *res*, i.e., a thing (as property) as opposed to a person, who has rights [4].

Having said that, in Italy, the purchase of animals is regulated by the civil code (article 1470 and subsequent). These articles regulate the purchase of real property, and they are also adopted in the field of the purchase of animals.

In actual fact, the only article specifically concerning animal purchase is article 1496 of the Italian civil code.

It states that the guarantee against vices in the sale of animals is regulated by special laws or by local usage or, if these are lacking, by article 1490 of the civil code and subsequent. The latter reads as follows: “The seller is obliged to guarantee that the object sold is free from vices which make it unsuitable for the usage to which it is destined or that appreciably decrease the its value.”

The buyer, within his/her rights may be able to exercise one of the two legal actions foreseen by the civil code, namely redhibitory action or estimatory action. “Redhibitory action” (*actio redhibitoria*) means rescission of sale and “estimatory action” (*actio estimatoria* or *quanti minoris*) means reduction of the price (article 1492 of civil code).

This can happen only when the animal is affected by a serious or chronic vice that is pre-existing and not easily recognizable. The time limits for rescission or exaction of proportionate reduction of the purchase price from the vendor under article 1495 of civil code are 8 days from the discovery of the defect and one year from the date of purchase.

In veterinary legal medicine, illness must be considered a vice in those cases where it makes an animal unsuitable for its specified use or significantly reduces its value [5].

An animal must be affected with a behavioral problem or an illness to be considered unsuitable for its specified use. An illness causes a disturbance in normal organic functioning, which may be localized or generalized, due to an anatomic alteration, and which will inevitably cause an appreciable impairment that could be permanent or temporary.

To qualify as vice, however, illness must be [5,6]:

1. Pre-existing or have a pre-existing cause
2. Hidden: This means that it cannot be discovered by an ordinary inspection or examination; or rather, is not easily recognized, at the moment of purchase it cannot be detected using the normal due diligence
3. Serious or chronic so as to affect the use of the animal or such that, if the buyer knew of the disease, he/she would not have entered into the contract. In fact, defect renders the thing sold unfit for the use for which it is intended, or diminishes its fitness for the intended use to such an extent that the vendee would not have bought it or would have given a lower price if he had been aware of the defect.

## Materials and Methods

### Ethical approval

This study was carried in accordance with the ethical principles of Veterinarians’ Ethical Code [7] and the Italian and European regulations on animal welfare [8].

### Animals and procedures

The medical records of n. 28 subjects, referred with suspected of congenital heart disease, were examined at a private veterinary clinic in Milan (Italy). All information was collected in accordance with the principles of correctness, lawfulness, transparency [9], protection of privacy, rights of the person concerned, and article 13 of Italian legislative decree no. 196/2003.

The subjects, aged from 3 to 24 months, reported history and symptoms related to heart disease (fatigue, exercise intolerance, syncope, pallor or cyanosis of the *mucosae*, detection of heart murmurs on auscultation on the chest).

All the animals underwent a complete examination of the cardiovascular system including chest X-ray for the evaluation of cardiac silhouette and blood vessels, while electrocardiogram and echocardiography were used to establish the presence of congenital malformations. Echocardiographic examination was carried out using the ultrasound system MyLab™ ClassC equipped with mechanical probes with a frequency of 2.5-3.5, 5-7.5, 7.5-10 MHz. The examination was in accordance with the standards proposed by the American Society of Echocardiography [10,11].

Angiocardiography was reserved for the animals that underwent percutaneous interventional procedures or when necessary for diagnostic purposes.

## Results

Cardiac abnormalities were detected in 26 dogs and 2 cats, 19 males and 9 females.

The animals belonged to various breeds. The canine breeds most frequently represented were boxer (n. 4), cavalier King Charles spaniel (n. 2), Chihuahua (2), French bulldog (n. 2), Labrador retriever (n. 2), and German shepherd dogs (n. 2).

The diagnosis included the presence of congenital heart disease alone or associated with other cardiac malformations (Table-1). Valvular alterations were found in 21 cases; pulmonary stenosis (PS) was present in 11 dogs, aortic stenosis in 4, and tricuspid valve dysplasia in 4, stenosis and mitral valve dysplasia in 1. Three animals showed septal defects, persistent patent ductus arteriosus (PDA) was identified in 5, tetralogy of fallot (TOF) in 2.

**Table-1:** Signalement and diagnosis of heart disease in the dogs examined.

Case	Species	Breed	Sex	Age	Diagnosis	Type of legal action	Discovery of the defect	The time when the seller was informed of the diagnosis of the hearth disease
1	Canine	West Highland Terrier	F	5 months	Pulmonic stenosis (type A)+Tricuspidal dysplasia	*Actio estimatoria*	<1 year from the date of purchase	<8 days from the discovery
2	Canine	Boxer	M	9 months	Pulmonic stenosis (type A)+Pulmonic insufficiency	*Actio redhibitoria*	<1 year from the date of purchase	<8 days from the discovery
3	Canine	Wirehaired Pointing Griffon	F	12 months	Tricuspidal dysplasia	*Actio redhibitoria*	<1 year from the date of purchase	<8 days from the discovery
4	Canine	Chihuahua	M	5 months	Interventricular defect+PDA	*Actio redhibitoria*	<1 year from the date of purchase	<8 days from the discovery
5	Canine	Chihuahua	M	24 months	TOF	*Actio redhibitoria*	<1 year from the date of purchase	10 days from the discovery
6	Feline	Exotic	M	12 months	PDA	*Actio estimatoria*	<1 year from the date of purchase	<8 days from the discovery
7	Canine	Dachshund	F	2 months	PDA	*Actio redhibitoria*	<1 year from the date of purchase	<8 days from the discovery
8	Canine	Maltese	M	4 months	PDA	*Actio estimatoria*	<1 year from the date of purchase	<8 days from the discovery
9	Canine	German shepherd Dog	F	10 months	Pulmonic stenosis (Type A)	*Actio estimatoria*	<1 year from the date of purchase	<8 days from the discovery
10	Canine	Boxer	M	4 months	Pulmonic stenosis (Type B)	(a)	(a)	(a)
11	Canine	German shepherd dog	M	12 months	Subaortic stenosis	*Actio redhibitoria*	<1 year from the date of purchase	<8 days from the discovery
12	Canine	Boxer	M	12 months	Pulmonic stenosis (Type A)+Subaortic stenosis	*Actio estimatoria*	<1 year from the date of purchase	<8 days from the discovery
13	Canine	Terranova	M	6 months	PDA with rightleft shunt	(a)	(a)	(a)
14	Canine	Labrador	F	5 months	Tricuspidal dysplasia	*Actio redhibitoria*	<1 year from the date of purchase	<8 days from the discovery
15	Canine	Pointer	M	3 months	Pulmonic stenosis (Type B)	*Actio redhibitoria*	<1 year from the date of purchase	<8 days from the discovery
16	Canine	Labrador	M	7 months	Tricuspidal dysplasia	(a)	(a)	(a)
17	Canine	Sheep dog	F	12 months	Subaortic stenosis	*Actio estimatoria*	<1 year from the date of purchase	<8 days from the discovery
18	Canine	Fox terrier	M	6 months	Mitralic stenosis+Aortic stenosis	*Actio estimatoria*	<1 year from the date of purchase	<8 days from the discovery
19	Canine	Cavalier King Charles Spaniel	M	10 months	Pulmonic stenosis (Type A)+Septal defect+PDA+cranial vena cava at left	*Actio estimatoria*	<1 year from the date of purchase	<8 days from the discovery
20	Feline	Persian	F	12 months	Atrioventricular channel	(a)	(a)	(a)
21	Canine	French Bulldog	M	8 months	Pulmonic stenosis (type A)	*Actio estimatoria*	<1 year from the date of purchase	<8 days from the discovery
22	Canine	Weimaraner	M	6 months	Mitral dysplasia	Reimbursement of medical expences	(a)	(a)
23	Canine	Golden Retriever	F	8 months	Pulmonic stenosis (type A)	(a)	(a)	(a)
24	Canine	Boxer	M	12 months	Subaortic stenosis+Pulmonic stenosis (type B)	(a)	(a)	(a)
25	Feline	Bernese	M	4 months	Perimembranous Interventricular defect with left-right shunt	(a)	(a)	(a)
26	Canine	Border collie	M	5 months	TOF	*Actio redhibitoria*	<1 year from the date of purchase	<8 days from the discovery
27	Canine	French Bulldog	F	3 months	Pulmonic stenosis	*Actio redhibitoria*	<1 year from the date of purchase	<8 days from the discovery
28	Canine	Cavalier King Charles Spaniel	M	3 months	PDA	*Actio redhibitoria*	<1 year from the date of purchase	<8 days from the discovery

(a): Any legal action was requested by owner. PDA=Patent Ductus Arteriosus, TOF=Tetralogy of Fallot

Subaortic stenosis (SAS) lesions were classified according to Pyle *et al*. [12] in relation to the severity and extent of the congenital defect within the endocardium in types 1, 2 and 3.

PS lesions were classified according to their appearance in Type A (presence of fused and/or thickened pulmonary flaps) and B (hypoplasia of pulmonary ostium associated with thickening and/or fusion of valve leaflets). The history varied with relation to the type or the severity of cardiac disease and included lethargy, anorexia, exercise intolerance, shortness of breath, slow growth and, in severe cases, collapse and/or syncope. The age of onset of symptoms ranged between 4 and 30 days. Cardiac murmurs had various locations, timing and quality according to the cardiac abnormalities.

The veterinarian informed the owners about the nature of the heart defect and the possibility of undertaking a redhibitory or estimatory action against the seller within 8 days from the discovery of the disease and within one year of purchase.

The Italian law on selling is well defined. Article 1490 of Italian civil code specifies that, in the presence of defects in the object sold, the only possible actions are contracted termination (so-called redhibitory action) and/or a reduction in price (so-called estimatory action), besides the request for compensation for the damage suffered (articles 1492 and 1494 of the Italian civil code).

The owners of cases no. 1, 6, 8, 9, 12, 17, 18, 19 and 21 opted for the litigant request, despite the owner of the cat (case no. 6) having carried out an act of property on animal (castration). In this case, the buyer has the obligation to return the animal in the same condition as at purchase.

In case no. 5, the defect was found during the warranty period prescribed by law (1 year from the sale). After certification of the disease, the owner returned the animal to the seller requesting redhibitory action. For an action for defect (i.e., TOF) to be eligible, it should be undertaken without delay by the owner or the buyer.

Indeed, pursuant to the article 1495 of the civil code, the buyer loses the warranty rights if the seller is not informed of presence of the defect within 8 days of its discovery, unless another deadline is established by the parties or by laws (Civil Cassation, Sec. II on August 30, 2000, no. 11452).

Case no. 11 involves a standard international property contract. The owner requested redhibitory action or contract termination, so as to return the animal to the seller. The seller was obliged to refund the full purchase price of animals and to pay costs incurred during the period, including the transfer of ownership.

Case no. 22 (a dog) died before legal action could be taken. In this case, the owner requested and obtained a reimbursement of medical expenses only.

In case no. 10, 13, 16, 20, 23, 24 and 25, the owners decided to have surgery on their animals pursuing no action against the seller.

In case no. 26, the owner requested termination of the contract of sale (sentence n. 9330 of 17.05.2004 of the supreme court) because the necropsy certified that the cause of animal death was congenital cardiac disease (TOF). In this case, it is essential for the owner to prove that the death of the animal did not come about from accidental causes but was caused by the existence of defect.

For case no. 28, redhibitory action was requested but the hair trichotomy necessary for echocardiographic examination was interpreted as property action on animal. The dog, therefore, remained with the buyer and only an estimatory action was allowed. This led to a partial reimbursement of the purchase price, commensurate with the reduced value that is a consequence of the defect. The aim of the action was “proportionally to maintain unchanged the initial equivalence ratio between the contract price and goods.” In the opinion of Rubino [13], it is not a form of partial termination of contract, but a limited and special form of damages. The rationale behind this is “the difference between the agreed price and the lower price that the buyer would have had to pay if he had known of the vices from the outset.”

In cases no. 2, 3, 4, 7, 14, 15 and 27, the puppies had been recently purchased and all the owners requested redhibitory action. The sellers accepted the return of the puppies to their original domicile and to pay the full reimbursement, which included all costs of diagnosis and possible therapy.

## Discussion

Congenital cardiac diseases represent a substantial cause of morbidity and mortality in dogs <1 year of age [14]. The defects most commonly reported in dogs and cats are PDA, PS and SAS, ventricular septal defects, tricuspid dysplasia, and TOF [15,16]. The exact prevalence of these abnormalities is difficult to determine because the clinical findings are varied and not easily recognizable. Some malformations do not cause audible cardiac murmurs or lead to perinatal death. Most dogs and cats affected are purebreds, with a higher prevalence in the German shepherd, bulldog, Labrador retriever, collie, boxer, Maine coon cat and Persian cat [14].

Congenital and/or hereditary heart diseases (e.g., persistent ductus, PS, aortic stenosis, etc.) fall into the category of defect, since they satisfy the requirements of pre-existence, hidden and causing an alteration of the use. Congenital heart disease, such as PS or patent ductus pervious, may be present and have clinical manifestation at birth depending on their severity. Since the commerce of puppies below 2 months of age is forbidden by law, puppies or kittens with serious PS or patent ductus pervious generally die in the postnatal period. Consequently, the lesions identified are of lesser or moderate severity. Other lesions, such as SAS, may be minimal at birth, but progressively worsen during the first 6-12 months of life. The diseases may not be identifiable by using normal care of bonus pater familias; in many cases, the symptoms are subtle and non-specific (weakness, fatigue, depressed sensorium, pale mucous membranes, ascites, etc.,) so can lead to misdiagnoses. They must be severe enough to affect the use of the animal or such that if the defect had been known, the contract would not have been concluded.

## Conclusion

In the cases examined, the congenital or hereditary diseases were serious enough to cause death. The defects detected may be considered a latent defect. For this reason, the seller should guarantee the presence of a certificate attesting the state of health. Furthermore, it would be desirable for the seller to seek the assistance of a veterinary cardiologist for congenital heart disease, to establish the health status of the animals and to avoid disputes between the parties (buyer and seller). It would also be desirable that the parties compile a private agreement (Box 1).

**BOX 1**

Private Agreement

Between the undersigned Mr./Miss …………………………., seller, and Mr./Miss ……………………….., buyer, it is agreed as follows. Mr/Miss …………… ……………. sells to the following animal: Canine species, breed …………………………………………………, Age…., sex …., Coat…………….., n microchip. ……………………….., Ensuring their eviction and also undertake to the buyer to purchase the property of the animal in question.

The buyer pays the seller the sum of EUR …………… as deposit / withdrawal engaging account of the animal within ……..; of this amount the seller shall issue a receipt with the release of this signature. That animal is healthy and delivered accompanied by a veterinary certificate attesting to the health of the animal performed by Dr. ……………. on………… The seller guarantees that the animal sold is free from defects, problems, and diseases that make it unsuitable for its intended use or to impair appreciably the value. This guarantee is valid for one year after delivery of the animal. After this deadline and in case of completions of property deeds not explicitly agreed and authorized by the seller that you will feel free from the obligations entered into with the signing of this letter. The purchaser agrees to keep the animal as the bonus pater familias with the obligation to notify the seller promptly any signs of discomfort of the animal.

Any dispute arising between the parties, subsequent to this agreement and in particular those regarding the interpretation, execution, performance, breach, termination of this contract, shall be settled by an arbitration panel composed of three members, two appointed by each party and the third by the first two arbitrators so appointed as aforesaid, and in case of disagreement by the President of the Court has territorial jurisdiction. In any case, the arbitration shall be decided in accordance with the fairness of the arbitration amicable manner without formal procedures and its decision will be final.

Date

The buyer (Signature)

The seller (Signature)

Until appropriate legislation regulating in detail the sale of pet animals is in place, it might be useful to require a temporal extension of warranty concerning hereditary defects.

In conclusion, the authors propose to formalize plans for the eradication of hereditary and/or congenital heart disease. These would primarily be based on ethical principles and would exclude genetic selection for certain characteristics, which may promote phenotyping of hereditary heart defects.

## Authors’ Contributions

AP and MP generated the concept, draft and revised the manuscript. AP, MP, VQ, NR, RB and BG collected materials. All authors read and approved the final manuscript.
